# m1A-Ensem: accurate identification of 1-methyladenosine sites through ensemble models

**DOI:** 10.1186/s13040-023-00353-x

**Published:** 2024-02-15

**Authors:** Muhammad Taseer Suleman, Fahad Alturise, Tamim Alkhalifah, Yaser Daanial Khan

**Affiliations:** 1 Department of Computer Science, School of Systems and Technology, University of Management and Technology, Lahore, 54770 Pakistan; 2 Department of Computer, College of Science and Arts in Ar Rass, Qassim University, Ar Rass, Qassim Saudi Arabia

**Keywords:** Respiratory Disease, Artificial Intelligence, Decision Trees, Statistical Model, Computational Model, Sequence Analysis, Genetics, Nucleotide Sequence, RNA, Computational Biology

## Abstract

**Background:**

1-methyladenosine (m1A) is a variant of methyladenosine that holds a methyl substituent in the 1st position having a prominent role in RNA stability and human metabolites.

**Objective:**

Traditional approaches, such as mass spectrometry and site-directed mutagenesis, proved to be time-consuming and complicated.

**Methodology:**

The present research focused on the identification of m1A sites within RNA sequences using novel feature development mechanisms. The obtained features were used to train the ensemble models, including blending, boosting, and bagging. Independent testing and k-fold cross validation were then performed on the trained ensemble models.

**Results:**

The proposed model outperformed the preexisting predictors and revealed optimized scores based on major accuracy metrics.

**Conclusion:**

For research purpose, a user-friendly webserver of the proposed model can be accessed through https://taseersuleman-m1a-ensem1.streamlit.app/.

## Introduction

**Fig. 1:**
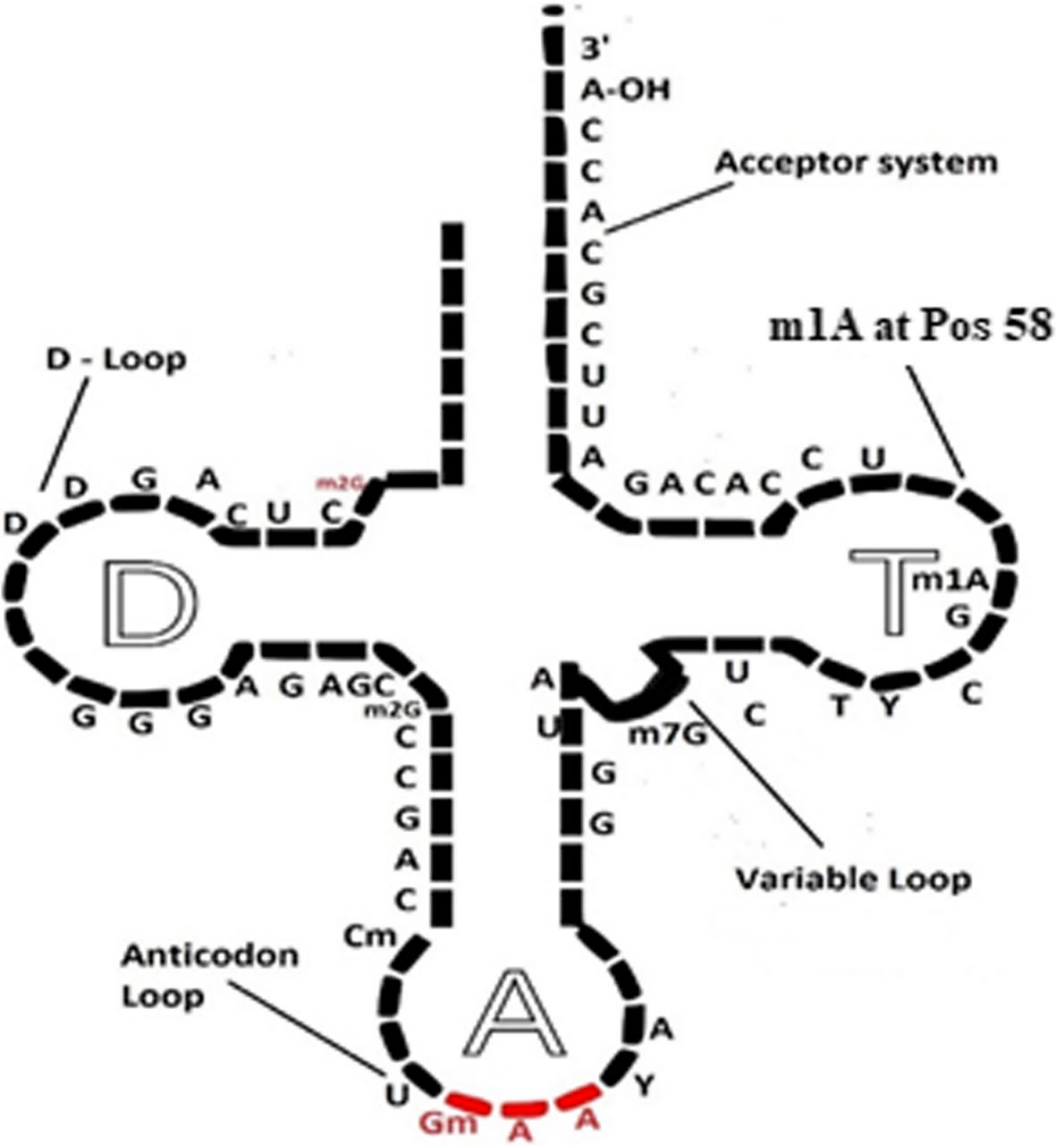
Position 58 in tRNA loop contains 1-methyladenosine site

1-methyadenosine (m1A) sites are reported to be present in transfer RNA (tRNA), messenger RNA (mRNA), and ribosomal RNA (rRNA). In tRNA, these sites occurred in T¥C loop at position 58, as shown in Fig. 1. The identification of m1A sites is significant because of its prominent role in various human diseases such as Mitochondrial respiratory chain defects, Neurodevelopmental regression, X-linked intractable epilepsy, and Obesity [1–3]. Moreover, this PTM modification is actively involved in protein translation, reverse transcription, and reticence in tumors. The m1A site prediction is critical for fully comprehending its potential functions. Site-directed mutagenesis and mass spectrometry have been proposed as methods for detecting m1A sites, although both are complex and time-consuming [4]. The availability of sequence-based datasets has increased the possibility of applying computational intelligence methods for the prediction of PTM sites.

Chen et al. [5] initially developed a predictor, RAMPred, for the identification of m1A sites using *Homosapiens*, *Mus musculus,* and *Saccharomyces cerevisiae* samples*.* The obtained RNA samples were encoded using nucleotide chemical property (NCP). The obtained features were used to train the support vector machine (SVM) based model. The results revealed 99.13% accuracy (*ACC*)*,* 99.89% specificity (*Sp*), 98.38% sensitivity (*Sn*), and a 0.98 Matthews correlation coefficient (*MCC*). The researchers also developed an online webserver for RAMPred. In another study, Chen et al. [6] developed a predictor, iRNA-3typeA, for the identification of three types of RNA methylation sites, including 6-methyladenosine (m6A), m1A, and adenosine-to-inosine (A-to-I). The same data samples of *Homosapiens* and *Mus Musculus* were used previously in RAMPred. The results revealed an accuracy score of 99.13% in *Homosapiens* and 98.73% in *Mus musculus* species. A 41 nucleotides lengthy sample was used, and cross validation test was carried out for performance evaluation. In another study Liu et al. [7] suggested a prediction model, ISGm1A, that extract 75 genomic-based features from the RNA sequences. Five machine learning models were trained and validated through independent testing and cross validation. Sun et al. [8] developed a deep learning framework, DeepMRMP, based on bidirectional gated recurrent unit (BGRU) for the identification of multiple RNA post transcriptional modified (PTM) sites in *Homosapiens*, *Mus Musculus* and *Saccharomyces Cerevisiae* species. One-hot encoding was used to encode the nucleotides within a sequence i.e. A = [1,0,0,0], C = [0,1,0,0], G = [0,0,1,0], U = [0,0,0,1]. The model revealed 70.5% *ACC,* 0.85 *Sn*, 0.95 *Sp* and 0.83 *mcc*.

**Fig. 2:**
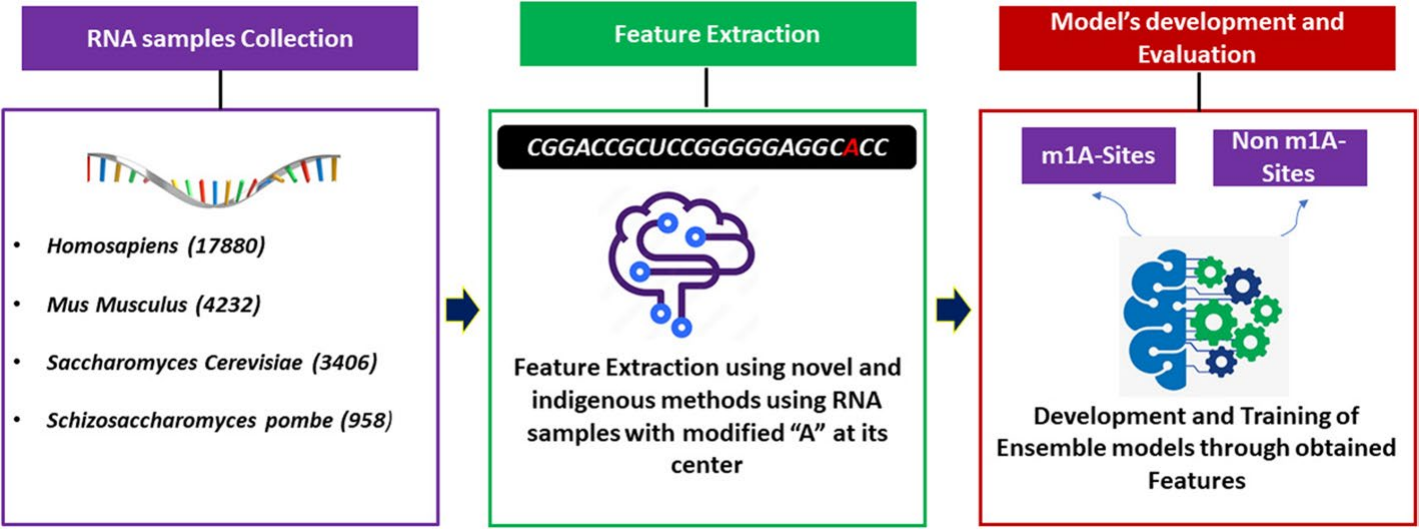
Current research methodology

Previous research studies dealt with the identification of m1A sites through traditional machine learning algorithms. However, such models are subjected to imbalanced data issue, overfitting and underfitting problems, and having limited context understanding. The current study proposed a novel framework for the prediction of m1A sites using ensemble models. These models were categorized into blending, bagging, and boosting which provides more rigorous training on dataset. It's worth mentioning here that RAMPred, iRNA3typeA, ISGm1A, and DeepMRMP have used the same dataset for training and validation. The dataset is composed of RNA sequences belonging to four species: *Homosapiens, Saccharomyces cerevisiae*, *Mus musculus* and *Schizosaccharomyces pombe*. The extraction of meaningful attributes from the sequences was carried out by considering the position and formation of nucleotide bases. Statistical moments were calculated that helped in feature dimensionality reduction in few metrics developed for attributes extraction. The performance of these ensemble models was evaluated through k-fold cross validation and independent set testing. The accuracy metrics such as *ACC*, *Sp*, *Sn,* and *MCC* were used to evaluate the ensemble models quantitatively. The results revealed that the proposed model outperformed in all accuracy metrics comparable to the preexisting m1A sites predictors. This research study was conducted in different phases, including benchmark dataset assortment, feature extraction and sample formulation, model development, training, and testing. Ultimately, a publicly accessible server was also made for facilitating in m1A sites detection. A methodology framework has been depicted in Fig. 2.

## Materials and methods

### Dataset collection

The dataset acquired from RMBase v2.0 [9] containing RNA samples from four species, including *Homosapiens*, *Saccharomyces cerevisiae*, *Mus musculus*, and *Schizosaccharomyces pombe* designated as *HS_17880*, *SC_3406*, *MM_4232* and *SP_958*. The dataset details have been mentioned in Table 1. After CD-Hit at 80%, the positive samples obtained were 11,978 and the negative samples obtained were 12,716. The cutoff was selected at 80% because of large number of samples. There might be a possibility of homology existing within samples. The window size for each RNA sample was chosen at 41 since this yielded the best overall performance. The window size was selected due the availability of 41nt verified samples and the optimized results revealed by this specific length. The m1A site-expressing RNA sample described in [1].

1 $$B\left(A\right)={B}_{-\intercal }{B}_{-\left(\intercal -1\right)}. . . . {B}_{-2}{B}_{-1}{AB}_{+1}{B}_{+2}. . . .{B}_{+\left(\intercal -1\right)}{B}_{+\intercal }$$

whereas “$A$” represents modified adenine of RNA sequences with methylated m1A sites.

**Table 1:** Details of RNA samples used in this study

Dataset	M1A_sites (positive)	Non-m1A sites (Negative)
*HS_17880*	8940	8940
*SC_3406*	1703	1703
*MM_4232*	2116	2116
*SP_958*	479	479

**Fig. 3:**
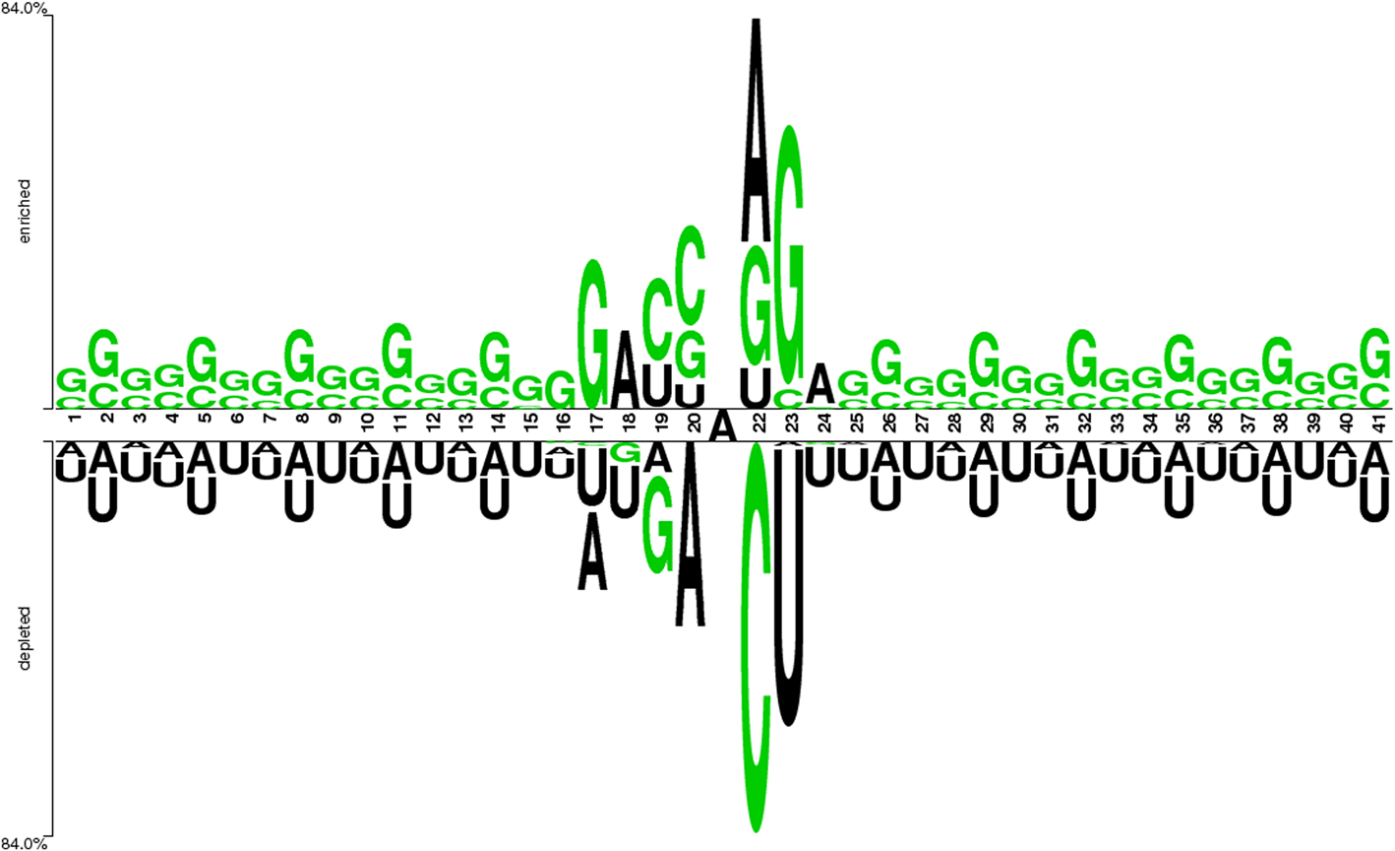
Two sample logos of the data samples representing nucleotide distributions

The arrangement of nucleotide bases within the acquired sequences can be visualized using a sequence logo. To achieve this, an online tool known as the "Two Sample Logo” was utilized. Figure 3 displays the sequence logo, which effectively represents the presence of cytosine (C), guanine (G), adenine (A), and uracil (U) within the dataset.

The nucleotides sample logo illustrates the concentration of “U” and “A” nucleotides throughout the sequence. However, the central position at “21” includes the “A”. Moreover, the nucleotide “G” is symmetrically distributed along the whole samples. It can be observed that “C” is only located from position 19 to 23 within nucleotide sequence.

### Feature extraction and development phase

The most important phase of computational procedures is feature extraction. During this stage, features are extracted to emphasize the dataset's unique characteristics [10]. Due to recent advances in information and data sciences, biotechnology has made major strides forward. Yet, the most difficult aspect is the development of computationally sophisticated models that transform raw biological input into counted, quantified vectors. Moreover, the loss of a single sequence or its associated properties must be prevented. This is due to the fact that all inputs to machine learning algorithms are vectors. The current research adopted a novel feature extraction method which includes various matrices and vectors for attaining the useful attributes from the sequences. These specialized vectors and matrices were indigenously developed for extracting divulged as well as concealed features within the sequences. This would be helping in developing more robust computational models that would assist in identification of m1A sites in an optimized way. To prevent the complete loss of the sequence-pattern information, Chou developed a pseudo-amino acid composition for proteins (PseAAC) [11]. Then pseudo-K-tuple nucleotide composition (PseKNC) was formulated as a result of the PseAAC success [12, 13]. Additionally, an RNA sequence, $X$, can be illustrated, as shown in [2].

2 $$X={X}_{1},{X}_{2},{X}_{3},\dots ,{X}_{i},\dots ,{X}_{n}$$

whereas,

$${X}_{i}\upepsilon \{C\left(cytosine\right), A\left(Adenine\right), G\left(guanine\right), U(uracil)\}$$

represents a nitrogenous base at a random position within an RNA sample. The genomic data used in this study was transformed into a matrix, $f{\prime}$, as shown in [3].

3 $$f{\prime}= {\left[{f}_{1}{f}_{2}{f}_{3}{f}_{4}\dots {f}_{ u}\dots {f}_{ \Omega }\right]}^{\intercal }$$

A single feature, ${f}_{u}$, depicts an arbitrary numerical coefficient which characterize a single feature. The transpose was taken for yielding discrete coefficients.

#### Statistical moments calculation

A fixed-length feature vector was computed from the genomic data using statistical moments [14]. Statistical moments are essential tools in statistics and probability theory that provide valuable information about the distribution of data. They are used to describe the shape, central tendency, spread, and other characteristics of a dataset. The significance of statistical moments lies in their ability to summarize and quantify various aspects of data distributions, making them useful in a wide range of applications, including data analysis, modeling, and decision-making. Moments of various distributions have been studied by analysts and mathematicians [15]. By computing the central, Hahn, and raw moments, a compact feature set was generated, which was then utilized to reduce the colossal input vector. Therefore, moments were computed for dimensionality reduction. The feature set was expanded to incorporate the scale and area of important moments to help differentiate between functionally distinct sequences. According to scientific investigations, genomic and proteomic sequence-based characteristics alter with the content and relative location of their bases [16]. Hence, the feature vector is best generated using mathematical and computational models that are sensitive to the relative location of component bases within genomic sequences. The features were transformed into compact coefficients that accurately reflect the data's mean and standard deviation using raw, central, and Hahn moments. While attempting to decipher a sequence, scale and position variations like the Raw and Hahn moments are preferable. $A two-dimensional matrix$, Ƕʹ, was built from the sequences, with each entry, Ƕ_*mn*_, representing the ${n}_{th}$ nucleotide base in the, ${m}_{th}$, sequence as expressed in [4].


4


Raw moments are used to derive location variant characteristics from extracted features [17]. Raw moments are described in [5], where the total number of raw moments is denoted by the value of u + v. The coefficients Ɲ_00_, Ɲ_01_, Ɲ_10_, Ɲ_11_, Ɲ_12_, Ɲ_21_, Ɲ_30_, and Ɲ_03_ were determined up to the third-degree polynomial [18, 19].

5 $${N}_{jk}= {\sum }_{c =1}^{m}{\sum }_{d =1}^{m}{c}^{j}{d}^{k}{\beta }_{cd}$$

The significance of the central moments is unrelated to the nucleotide’s location. These, on the other hand, are associated with the composition and form of the distribution [20]. Moreover, the central moments are associated with the nucleotides’ composition and distribution. For the current study, the central moments were computed and expressed in [6] as follows.

6 $${n}_{ij}= {\sum }_{b =1}^{n}{\sum }_{q =1}^{n}{\left(b- \mathcal{x}\right)}^{i}{\left(q- \mathcal{y}\right)}^{j}{\beta }_{bq}$$

Orthogonal moments are often preferred because they can represent data with the least amount of redundant information. Yet, even if the original sequences have been drastically shortened to a fixed length, the predictor still gets the effect of the whole sequence of data within the reduced feature vector due to the reversible quality of these moments. Hahn polynomials can be written as follows:

7 $${h}_{n}^{u,v}\left(r,N\right)= {\left(N+V-1\right)}_{n}{\left(N-1\right)}_{n}\times {\sum }_{k=0}^{n}{\left(-1\right)}^{k}\frac{{\left(-n\right)}_{k}{\left(-r\right)}_{k}{\left(2N+u+v-n-1\right)}_{k}}{{\left(N+v -1\right)}_{k}{\left(N-1\right)}_{k}}\frac{1}{k!}$$

where,$(u,v)$, are adjustable parameters that control polynomial shapes. Given a sequence in the form of a two-dimensional matrix, $M X M$, the Hahn moment can be described as mentioned in [8].

8 $${H}_{ij}= {\sum }_{q=0}^{N-1}{\sum }_{p=0}^{N-1}{\beta }_{ij}{h}_{j}^{\widetilde{u,} v}\left(p,N\right), m,n=\mathrm{0,1},N-1$$

#### Position Relative Incidence Matrix (PRIM)

The position relative incidence matrix (PRIM) was used to represent the relative positioning of nucleotide bases within an RNA sample [21]. The matrix, ${{\varvec{E}}}_{{\varvec{P}}{\varvec{R}}{\varvec{I}}{\varvec{M}}}$ [9], is a $4\mathrm{X }4$ matrix that represents any single nucleotide, ${{\text{V}}}_{{\varvec{m}}}$, at position $"{\varvec{m}}"$, with respect to other nucleotides within a sequence. The matrix generated 16 unique coefficients.

9 $${E}_{PRIM}= \left[\begin{array}{ccc}{V}_{A\to A}& {V}_{A\to G}& \begin{array}{cc}{V}_{A\to U}& {V}_{A\to C}\end{array}\\ {V}_{G\to A}& {V}_{G\to G}& \begin{array}{cc}{V}_{G\to U}& {V}_{G\to C}\end{array}\\ \begin{array}{c}{V}_{U\to A}\\ {V}_{C\to A}\end{array}& \begin{array}{c}{V}_{U\to G}\\ {V}_{C\to G}\end{array}& \begin{array}{c}\begin{array}{cc}{V}_{U\to U}& {V}_{U\to C}\end{array}\\ \begin{array}{cc}{V}_{C\to U}& {V}_{C\to C}\end{array}\end{array}\end{array}\right]$$

where, ${{\text{V}}}_{i\to j}$, represents the relative positioning of an arbitrary nucleotide base with respect to any other random base within a sequence. The occurrence of nucleotide base pairs (i.e., AA, AG, AU, …, CG, CU, CC) is significant in the feature extraction process. The formation of a $16\mathrm{ X }16$ matrix known as $\check{U}_{PRIM}$ [10], which results in 256 coefficients, was used to consider the frequency with which these base pairings occur in comparison to one another.

10 $${\check{U}}_{PRIM}= \left[\begin{array}{ccccccc}{\check{U}}_{AA\to AA}& {\check{U}}_{AA\to AG}& {\check{U}}_{AA\to AU}& \cdots & {\check{U}}_{AA\to j}& \cdots & {\check{U}}_{AA\to CC}\\ {\check{U}}_{AG\to AA}& {\check{U}}_{AG\to AG}& {\check{U}}_{AG\to AU}& \cdots & {\check{U}}_{AG\to j}& \cdots & {\check{U}}_{AG\to CC}\\ {\check{U}}_{AU\to AA}& {\check{U}}_{AU\to AG}& {\check{U}}_{AU\to AU}& \cdots & {\check{U}}_{AU\to j}& \cdots & {\check{U}}_{AU\to CC}\\ \vdots & \vdots & \vdots & \vdots & \vdots & \vdots & \vdots \\ {\check{U}}_{GU\to AA}& {\check{U}}_{GA\to AG}& {\check{U}}_{GU\to AU}& \cdots & {\check{U}}_{GA\to j}& \cdots & {\check{U}}_{GA\to CC}\\ \vdots & \vdots & \vdots & \vdots & \vdots & \vdots & \vdots \\ {\check{U}}_{N\to AA}& {\check{U}}_{N\to AG}& {\check{U}}_{N\to AU}& \cdots & {\check{U}}_{N\to j}& \cdots & {\check{U}}_{N\to CC}\end{array}\right]$$

Similarly, another matrix, Ƚ_*PRIM*_ [11], was formed for the tri-nucleotide base combination (i.e., AAA, AAG, AAU, …. CCG, CCU, CCC). A total of 4096 coefficients were yielded by this matrix. The central, Hahn and raw moments were computed for ${E}_{PRIM}$, $\check{U}_{PRIM}$ and Ƚ_*PRIM*_, that resulted in forming coefficients up to order 3.


11


#### Reverse Position Relative Incidence Matrix (RPRIM)

The primary objective of determining feature vectors is to collect as much relevant information as possible to develop an accurate prediction model. Reversing the sequence order yielded a reverse position relative indices matrix (RPRIM) in an effort to extract more information contained within the sequences [22]. Similarly with PRIM matrices, RPRIM was calculated using mononucleotide, dinucleotide, and trinucleotide combinations. For this reason, Ʀ_*RPRIM*_ was computed according to [12].


12


#### Frequency vector determination

The sequence's positional and compositional information is crucial in developing a feature set [23, 24]. The composition of the sequence can be determined by counting the frequency of each nucleotide. A frequency vector (Ᵹ) is used to store the count for each nucleotide or nucleotide pair in the sequence, and the method for calculating this vector has been described in [13].13where,  , is the count of the ${i}_{th}$ nucleotide in a sequence.

#### Generation of Accumulative Absolute Position Incidence Vector (AAPIV)

The AAPIV (accumulated information of individual nucleotide bases) is a method used to provide information on the frequency of each individual nucleotide base in a sequence [25]. This method is responsible for collecting and accumulating data related to the occurrence of each nucleotide base, including single and paired nucleotide bases [26, 27]. To achieve this, three different AAPIV vectors were generated, each representing a different level of granularity. These vectors were given the names ${S}_{AAPIV4}$ [14], ${S}_{AAPIV16}$ [15] and ${S}_{AAPIV64}$ [16]. Each vector represents a different level of granularity, with ${S}_{AAPIV4}$ containing information on four nucleotides, ${S}_{AAPIV16}$ containing information on sixteen nucleotides, and ${S}_{AAPIV64}$ containing information on sixty-four nucleotides. These vectors provide a useful tool for analyzing the composition of nucleotide sequences and can be used in a variety of biological applications. 


14



15



16


 where, þ_i,_ can be calculated as provided in [17].

17 $${\delta }_{i}={\sum }_{k=1}^{n}{{\text{p}}}_{k}$$

#### Reverse Accumulative Absolute Position Incidence Vector (RAAPIV) Generation

To analyze the reversed sequences, a reverse accumulative absolute position incidence vector (RAAPIV) had been devised in the research. Specifically, it involves reversing the order of the nucleotide sequences in order to gain a different perspective on the underlying data. There are three types of nucleotide combinations that were examined using the RAAPIV: single nucleotide combinations, di-nucleotide combinations, and tri-nucleotide combinations. The vector length for each of these combinations differs, with a length of 4 for single nucleotides, 16 for di-nucleotides, and 64 for tri-nucleotides. The expression (18), (19) and (20) referred to the combination of single nucleotide, dinucleotides and trinucleotides respectively. Overall, this technique provides a way to gain new insights into genetic sequences by analyzing them from a different perspective.

18 $${J}_{RAAPIV4}=\left\{{j}_{1,}{j}_{2,}{j}_{3,}{j}_{4}\right\}$$

19 $${J}_{RAAPIV16}=\left\{{j}_{1,}{j}_{2,}{j}_{3,}\dots ,{j}_{16}\right\}$$

20 $${J}_{RAAPIV64}=\left\{{j}_{1,}{j}_{2,}{j}_{3,}\dots ,{j}_{64}\right\}$$

#### Feature vector formulation

The outcome of the feature extraction operation was the creation of a single feature vector. This feature vector was then utilized as a prediction model input with 522 distinct values collected by PRIM, RPRIM, FV, AAPIV, and RAAPIV. Each feature vector in the dataset represents an individual sample. For binary classification, positive samples were labelled as "1" and negative samples as "0″ [28, 29]. Table 2 contains the detail of the number of features obtained from each vector or matrix individually.

**Table 2:** Number of features obtained from each vector and matrix

Vector/Matrix	Features obtained (Dimensions)
PRIM (${E}_{PRIM}$, $\check{U}_{PRIM}$, Ƚ_*PRIM*_)	90
RPRIM (Ʀ_RPRIM_)	90
Frequency Vector	84
AAPIV (${S}_{AAPIV4}$, ${S}_{AAPIV16}$, ${S}_{AAPIV64}$)	84
RAAPIV (${J}_{RAAPIV4}$, ${J}_{RAAPIV16}, {J}_{RAAPIV64}$)	84
two-dimensional matrix, Ƕʹ	90
**Total**	**522**

### Ensemble models development and training

Ensemble methods have gained popularity in the field of machine learning due to their enhanced prediction capabilities as compared to conventional single-model approaches [30, 31]. These methods combine the strengths of multiple models to achieve better overall performance, and they can be broadly classified into parallel and sequential methods. To address real world challenges, ensemble models help in building trust, model aggregation, prediction on different patterns based on diverse classifiers and features-based analysis. Parallel ensemble methods, such as bootstrap aggregation (or bagging), involve training multiple models concurrently on different subsets of the data. Sequential ensemble methods, on the other hand, involve training models sequentially, with each subsequent model learning from the errors of the previous one. Ensemble-based classification has been reported in various research studies. Akbar et al. [20] devised a novel method for the identification of anticancer peptides based on the genetic algorithms-based ensemble models which achieved optimized accuracy scores. Moreover, in another research study, authors devised an ensemble-based model for the identification of antitubercular peptides and the accuracy scores reported to be more than 90% [32]. Ahmed et al. [33] proposed, iAFPs-EnC-GA, an ensemble learning based model for the identification antifungal peptides. In the context of the investigation mentioned, three distinct ensemble models were applied including blending, bagging, and boosting.

#### Blending ensemble

Blending is an ensemble technique that combines the outputs of multiple classification or regression models using a meta-classifier or meta-regressor [34, 35]. In this approach, the base-level models are first trained, and their outputs are then used as features for the meta-model. This meta-model leverages the knowledge of the base models to make more accurate and robust predictions. The current investigation employed four base models, including an artificial neural network (ANN), a k-nearest neighbor (KNN), a support vector machine (SVM), and a decision tree (DT). The gradient boost classifier was chosen as the meta-classifier to combine the outputs of these base models. Hyperparameter optimization is an essential step in machine learning, as it ensures that each model performs at its best. Table 3 presents the details of the hyperparameter optimization process for all the classifiers used in the blending ensemble deployment.

**Table 3:** Parameters tuning of the blending ensemble model

Base models	ANN	KNN	SVM	DT
Hyper-Parameters value(s)	*Hidden_layer_sizes* = 5,2 *Random_state* = 1 *Activation* = relu *Solver* = lbfgs *Learning rate* = adaptive *Alpha* = 0.0001	k = 3	*C* = 10 *Gamma* = 0.0001 *Kernel* = rbf *Coefficient* = 0.0 *Probability* = ‘True’ *Verbose* = ‘False’ *Random_state* = none	*Splitter* = ‘random’ *Max_depth* = 80 *min_samples_leaf* = 4 *random_state* = None
Meta classifier & its Hyper-parameter value(s)	Gradient Boost classifier *n_estimators* = 100, *criterion* = ‘mse’			

#### Bagging ensemble

The bagging ensemble methods in the research deployed in such a way that the trained samples were divided into smaller subsamples for the base models using a subsampling approach with replacement and row sampling. This strategy ensures that each base model is trained on a different subset of the data, promoting diversity among the individual models and reducing the overall variance of the ensemble [36].

The test data were evaluated using the trained base models, and the final forecast was obtained through a voting mechanism, which typically involves majority voting for classification tasks or averaging for regression tasks. Four bagging models, namely the bagging classifier, random forest, extra tree, and decision tree classifier, were developed and trained as part of the investigation. For improved results, all the bagging classifiers were subjected to hyperparameters optimization. The hyperparameters such as number of trees (*n_estimators*), depth of each tree (*max_depth*), maximum features *(max_features*), and a few other important parameters such as *min_samples_split*, *bootstrap*, and *min_samples_leaf* were considered. Table 4 contains the hyper-parameter optimization information of the aforementioned bagging models.

**Table 4:** Parameters tuning of the bagging ensemble models

Bagging models	Random Forest	Extra tree classifier	Decision Tree classifier	Bagging classifier
Hyper-Parameter value(s)	*n_estimators* = 200 *max_depth* = 50 *max_features* = ‘Auto’ *min_samples_split* = 10 *min_samples_leaf* = 5	*n_estimators* = 100 *max_depth* = 40 *max_features* = ‘Auto’ *Bootstrap* = bool	*Splitter* = ‘random’ *Max_depth* = 80 *min_samples_leaf* = 4 *random_state* = ‘None’ *min_weight_fraction_leaf* = 0.1	*Base_estimator* = ‘DecisionTreeClassifier’ *N_estimators* = 100 *Oob_score* = ‘True’ *Random_state* = 0

#### Boosting ensemble

**Fig. 4:**
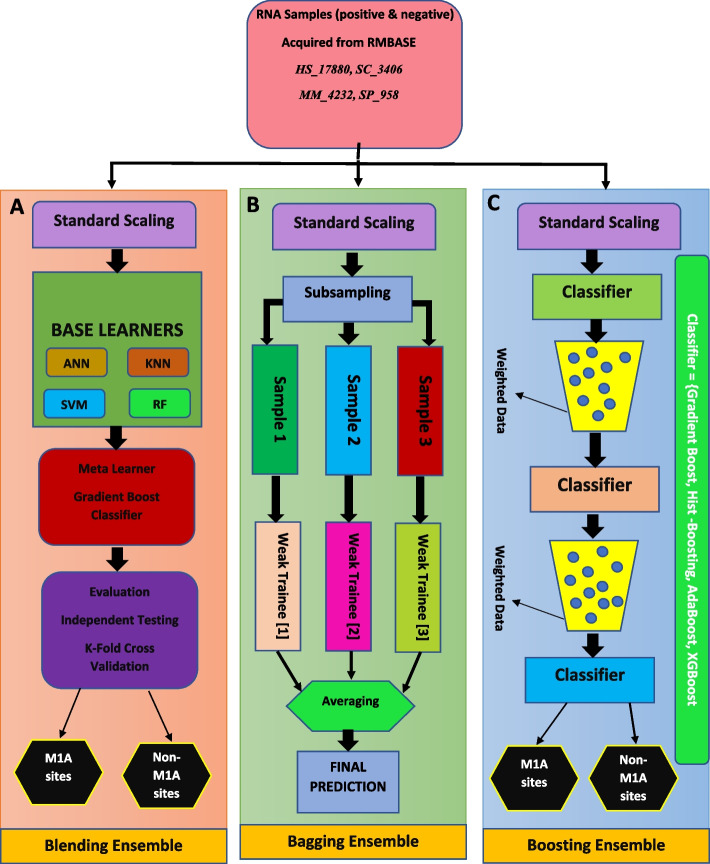
Ensemble models Development and Training/Testing for the Current research study using RNA samples from RMBase (**A**). Blending Ensemble (**B**). Bagging Ensemble (**C**). Boosting Ensemble

The boosting ensemble approach is designed to optimize the model based on the output of the preceding model in the sequence. It operates sequentially, with each model focusing on reducing the differentiable loss by learning from the errors of the previous model. This process helps boost the overall performance of the ensemble by combining the strengths of multiple weak learners. In the current investigation, several boosting ensemble training approaches were employed, including gradient boosting, histogram-based gradient boosting (HGB), AdaBoost, and extreme gradient boosting (XGB). To optimize the performance of the boosting ensemble models, various hyperparameters were fine-tuned, as shown in Table 5. Figure 4 depicts the concept diagram of ensemble model implementation for the current research study, which includes blending, boosting, and bagging.

**Table 5:** Hyper-parameters optimization of the boosting ensemble models

Boosting ensemble models	Gradient Boost	Hist-Boost	Adaboost	XGB
Hyper-Parameter value(s)	*learning_rate* = 0.1 *n_estimators* = 100 *criterion* = ‘mse’	*max_iter* = 200 max_depth = 40 *warm_start* = ‘True’	*Base_estimator* = ‘Gradientboostclassifier’ *n_estimators* = 50 *random_state* = ‘None’ *min_weight_fraction_leaf* = 0.1	*max_iter* = 100 *max_depth* = 40 *random_state* = 0

## Results and discussion

The trained models were subjected to validation using independent set testing and tenfold cross validation. The independent test was carried out using the standard “Train-Test” split method. However, tenfold cross validation is a rigorous test that divides the whole dataset into subsamples, where one sample is subjected to testing while the other nine are used for training. Different accuracy metrics were used to score the performance of all ensemble models, including *ACC*, *Sp*, *Sn*, and *MCC*.

### Metrics for evaluation

In this research, four metrics, ${S}_{n}$, ${S}_{p}$, $Acc$, and $MCC$ were used to evaluate the prediction models [37, 38]. The effectiveness of a categorization model may be measured in terms of its $Acc$. The $Acc$ rate is the ratio of the model's correct predictions to the total number of forecasts. It is the fraction of the dataset that was properly predicted relative to the total number of occurrences. Whereas Specificity $({S}_{p})$ is a metric used to evaluate the performance of a binary classification model, particularly in cases where the negative class is of greater importance. It measures the proportion of true negatives (TN) that are correctly identified by the model out of all negative instances. Sensitivity $({S}_{n})$ is a metric used to evaluate the performance of a binary classification model, particularly in cases where the positive class is of greater importance. It measures the proportion of true positives (TP) that are correctly identified by the model out of all positive instances. Matthews Correlation Coefficient $(MCC)$ is a metric used to evaluate the performance of a binary classification model, particularly when the classes are imbalanced. MCC takes into account the number of true and false positives and negatives to give a balanced measure of the model's performance. The accuracy metrics equations have been mentioned in [22].

21 $$\left\{\begin{array}{c}{S}_{n}=\frac{{\text{TP}}}{{\text{TP}}+{\text{FN}}} 0\le {S}_{n}\le 1\\ {S}_{p}=\frac{{\text{TN}}}{{\text{TN}}+{\text{FP}}} 0\le {S}_{p}\le 1\\ Acc=TP+TN / (TP+FP+FN+TN) 0\le Acc\le 1 \\ MCC=\left({\text{TP}}*{\text{TN}}-{\text{FP}}*{\text{FN}}\right) / \sqrt{({\text{TP}}+{\text{FP}})({\text{TP}}+{\text{FN}})({\text{TN}}+{\text{FP}})({\text{TN}}+{\text{FN}})} -1\le MCC\le 1\end{array}\right.$$

The TP denotes the m1A sites, whereas the TN denotes the non-m1A sites. A similar notation, FN, represents the total number of modified sites that were indeed actual m1A sites but were misidentified as false m1A sites. Furthermore, FP stands for the total number of false m1A sites that were misidentified. However, it's important to note that the measurements only apply to systems with a single class [39]. The false positive and false negative value have crucial roles in the performance evaluation of the system. A wrong detection of false positive leads to the wrong m1A site detection within a given RNA sample. Similarly, the increase in false negatives may result into the increase in non-m1A sites abnormally.

### Data preprocessing

The obtained feature set was subjected to data preprocessing by using standard scaling of sklearn preprocessing [40]. All the missing values were removed using standard scaling before input to the machine learning model.

### Independent set testing

**Fig. 5:**
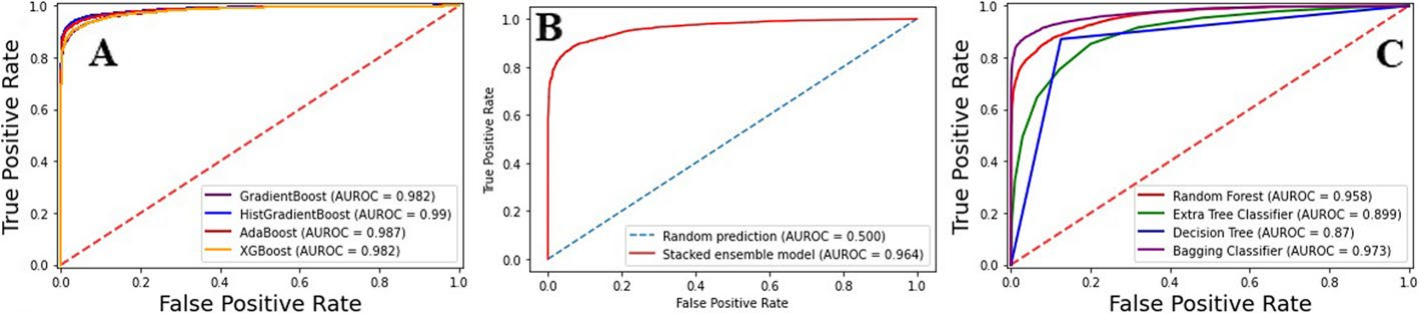
ROC curve of independent testing (**A**) Boosting Ensemble (**B**) Blending Ensemble (**C**) Bagging Ensemble

An Independent test set was carried out to validate all the ensemble models, including blending, bagging, and boosting. The independent set was created using the standard “train-test split” method with a 70% training and 30% testing dataset [41, 42]. There were 8385 positive and 8901 negative train samples. The test samples were 3593 positives and 3814 negatives. It is important to mention that training and test samples were separate frofutm each other. Table 6 contains the results revealed by all the ensemble models deployed for the current research. Whereas Fig. 5 depicts the area under curve (AUROC) of the ensemble model in independent testing.

**Table 6:** Independent testing result

Model		*Acc*	*S*_*p*_	*S*_*n*_	*MCC*	*F1-score*	*AUROC*
**Bagging**	*Random Forest*	0.88	0.93	0.85	0.77	0.87	0.95
	*Extra Tree Classifier*	0.81	0.87	0.75	0.63	0.80	0.89
	*Decision Tree*	0.87	0.87	0.87	0.74	0.86	0.87
	*Bagging classifier*	0.92	0.97	0.86	0.84	0.91	0.97
**Boosting**	*Gradient Boost*	0.93	0.97	0.89	0.87	0.93	0.98
	*HGB*	0.99	0.98	0.97	0.98	0.97	0.99
	*AdaBoost*	0.94	0.97	0.92	0.89	0.94	0.98
	*XGBoost*	0.93	0.97	0.93	0.87	0.93	0.98
**Blending**		0.91	0.86	0.94	0.81	0.90	0.96

### 10-Fold cross validation

The cross-validation approach is used to test all the samples while splitting the dataset into “k” disjoint folds [43, 44]. The robustness of a model is demonstrated by this more stringent test. In this test, k-1 folds (partitions) were trained on the model, while testing was performed on the left-over fold [45]. The test was repeated 10 times due to the number of folds used in this study, i.e., k = 10. Cross-validation results have been listed in Table 7.

**Table 7:** 10-Fold cross validation results

**Model**		***Acc***	***S***_***p***_	***S***_***n***_	***MCC***	***F1-score***	***AUROC***
**Bagging**	*Random Forest*	0.82	0.85	0.81	0.77	0.80	0.86
	*Extra Tree Classifier*	0.91	0.92	0.93	0.89	0.83	0.94
	*Decision Tree*	0.87	0.87	0.87	0.74	0.86	0.87
	*Bagging classifier*	0.94	0.98	0.88	0.86	0.93	0.98
**Boosting**	*Gradient Boost*	0.90	0.94	0.87	0.85	0.92	0.96
	*HGB*	0.98	0.97	0.95	0.97	0.96	0.98
	*AdaBoost*	0.99	0.98	0.96	0.97	0.96	0.99
	*XGBoost*	0.92	0.96	0.92	0.86	0.92	0.97
**Blending**		0.91	0.93	0.85	0.92	0.82	0.93

Several statistical tests were conducted to verify the effectiveness of the ensemble models implemented in this study. The primary goal of these tests was to compare the performance of various learning algorithms in achieving accurate classification outcomes. One of the tests conducted was a two-proportion test, commonly referred to as the Z test, on the ensemble models. This Z test was utilized to assess whether there existed a significant distinction between the two sets of samples. To establish such a distinction, the critical value (*p*) needed to be below 0.05, indicating the rejection of the null hypothesis. Furthermore, a resampled paired t-test was employed, using a predetermined set of trials, to measure the accuracy of the algorithms. McNemar's test, another statistical test, was applied to evaluate the significance of the difference between two proportions in a 2 × 2 contingency table. The resulting "*p*" values from these tests are listed in Table 8.

**Table 8:** Statistical test results of blending, boosting and bagging ensemble models

Model		*Z-test*	*Resampled paired t-test*	*McNemar’s test*
**Bagging**	*Random Forest*	0.00156	0.00089	0.0017
	*Extra Tree Classifier*	0.00162	0.00052	0.0019
	*Decision Tree*	0.00137	0.00059	0.0033
	*Bagging classifier*	0.00130	0.00090	0.0087
**Boosting**	*Gradient Boost*	0.00144	0.00090	0.0080
	*HGB*	0.00170	0.00075	0.0069
	*AdaBoost*	0.00149	0.00055	0.0049
	*XGBoost*	0.00129	0.00034	0.0038
**Blending**		0.00159	0.00045	0.0029

**Fig. 6:**
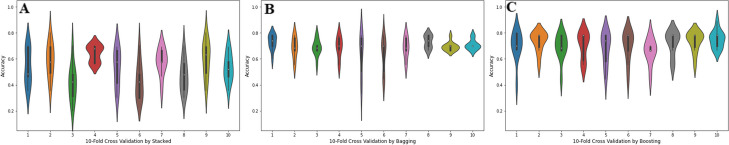
Violin plots of 10-Fold cross validation accuracy (Acc) metric results for (**A**) Blending ensemble (**B**) Bagging ensemble and (**C**) Boosting ensemble

The violin plot is a graphical representation that combines elements of a box plot and a kernel density plot to display the distribution of numerical data for one or more groups [46]. It uses density curves to illustrate the probability density of the data at different values, giving a clear visualization of the data distribution, including its central tendency, dispersion, and shape. Key elements of a violin plot include (1) a central white dot representing the median of the data, which indicates the middle value when the data is sorted in ascending order. (2) A black bar in the middle of the violin, showing the interquartile range (IQR), which represents the spread of the middle 50% of the data. () Dark black lines extending from the black bar to the lower and higher neighboring values, indicating the range of the data within 1.5 times the IQR from the lower and upper quartiles. Figure 6 displays the violin plots for the accuracy values obtained in each fold for the best ensemble models in the blending, bagging, and boosting categories.

**Fig. 7:**
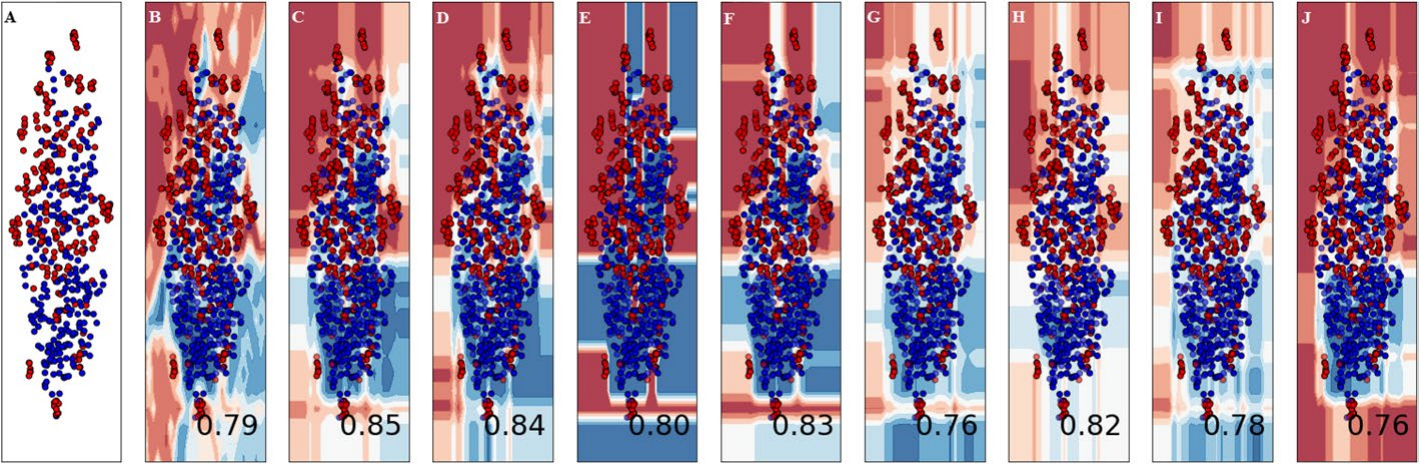
Boundary visualization of ensemble models used in this study as follows: (**A**). Input data **(B**). Blending (**C**). Random Forest (**D**) ExtraTree (**E**) Decision Tree (**F**) Bagging (**G**) Gradient Boost (**H**) Histo Gradient Boost (**I**) Adaboost (**J**) XGBoost

The application of supervised machine learning models can prove beneficial in various categorization tasks. Nonetheless, relying solely on numerical predictions might not be enough. Gaining a comprehensive understanding of the actual decision boundary that delineates the different groups is crucial. Consequently, the classification algorithms employed in this research were examined using a decision surface to enhance their accuracy. A decision surface map is a visual representation where a trained machine learning system predicts a coarse grid covering the input feature space. This method allows for a better understanding of the model's decision-making process by illustrating the regions in which the model assigns a particular class to input data points. Figure 7 displays the decision surface plots of the classification algorithms used in this research. By examining these plots, one can gain insights into how the algorithms differentiate between the various classes and the effectiveness of their decision-making process. This information can be valuable for refining the models, improving their accuracy, and ensuring more reliable outcomes in categorization tasks.

### Comparison with preexisting predictors

The proposed model was built on the best performing HGB ensemble model and compared with preexisting predictors to assess the model’s efficacy on the independent datasets. The predictors were RamPred, Deepmrmp, irna3typeA, and ISGm1A. It was observed that the proposed model, m1A-Ensem, outperformed exhibiting 0.99 *ACC*, 0.98 *Sp*, 0.97 *Sn,* and 0.98 *MCC*. The comparative results have been mentioned in Table 9. The use of vectors and matrices helped in extracting obscured features within the sequences. Moreover, the hyperparameter optimization of ensemble models helped in gaining promising accuracy scores. The identification of m1A sites is vital as this RNA modification has been implicated in various diseases such as Mitochondrial respiratory chain defects, Neurodevelopmental regression, X-linked intractable epilepsy, and Obesity. Moreover, m1A sites help in gene regulation procedures such as gene splicing, RNA stability and regulatory mechanisms. This modification is also involved in RNA folding and structure stability. Detecting these sites accurately is a critical step towards understanding the mechanisms behind these diseases and developing effective biomarkers for drug discovery. To address this issue, researchers have developed a comprehensive strategy that involves feature development and representation, merging multiple computational models, and testing the model using a variety of methodologies. This approach has resulted in the creation of a predictive model that outperforms existing models in identifying m1A sites. Extensive trials have shown that the proposed model has a high degree of precision, resilience, and scalability. Its accuracy in identifying modified m1A sites has been demonstrated through various testing methodologies, indicating its potential usefulness in research. Overall, the development of this predictive model represents a significant advancement in the field of RNA modification research, providing a valuable tool for researchers and clinicians in their efforts to better understand and treat diseases associated with m1A sites.

**Table 9:** Comparison with preexisting predictors

Model	Independent set test			
	**Acc (%)**	**S**_**p**_	**S**_**n**_	**MCC**
RAMPred	98.73	0.99	0.95	0.96
irna-3typeA	84.6	0.93	0.88	0.91
Deepmrmp	70.5	0.95	0.85	0.83
ISGm1A	83.5	0.83	0.83	0.67
m1A-Ensem	99.9	0.98	0.97	0.98

### Limitations and future work

The limitation of the current work is the availability of RNA samples from a few species only. The number of available samples also limits the possibility of training computational models. Moreover, the discovery of new m1A sites related to samples will require the development of new models and training of those models on latest data samples. This will be affecting the results obviously. Moreover, the scope of the study is limited to the development of ensemble models for the identification of m1A sites. The prediction of m1A sites through deep learning models using the available data samples can be attempted in the future.

## Web server availability

**Fig. 8:**
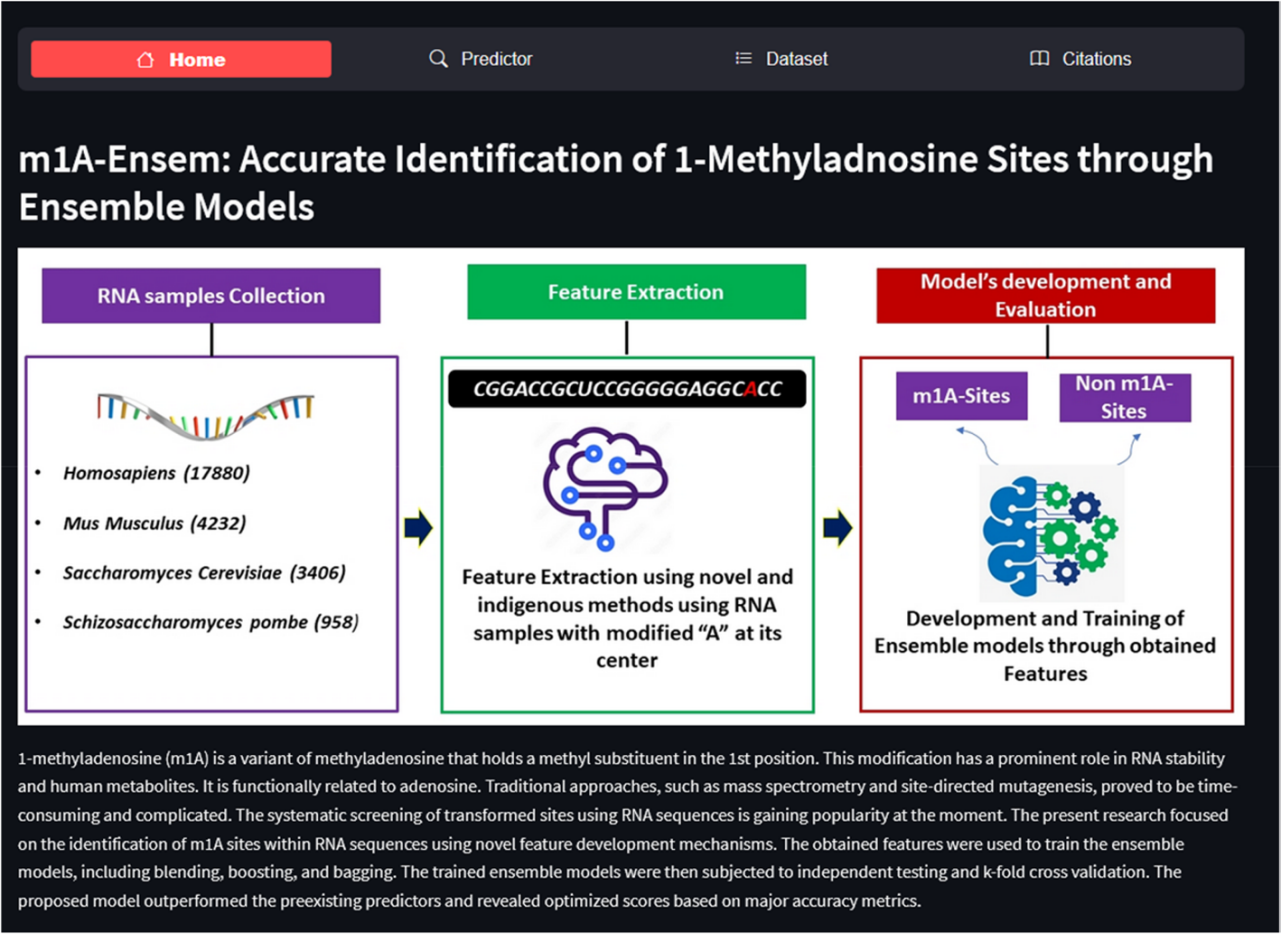
Screenshot of m1A-Ensem prediction webserver

**Fig. 9:**
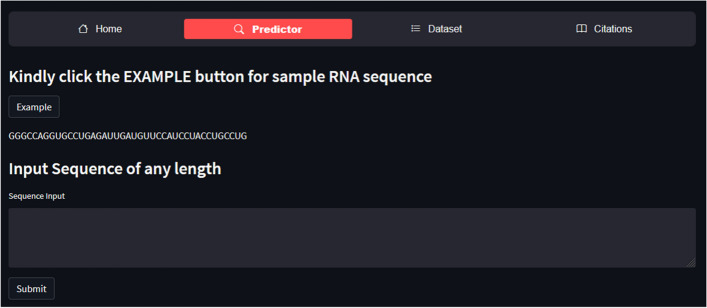
Image showing webserver “Predictor” page with “Example” sequence

**Fig. 10:**
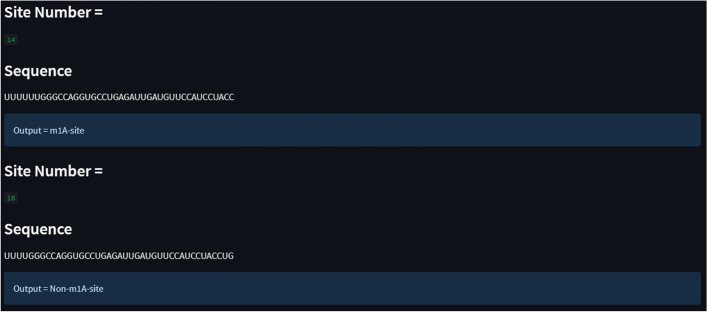
Webserver identifying m1A and non-m1A sites within RNA sample

**Fig. 11:**
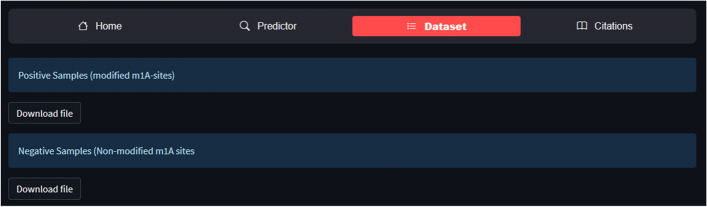
“Dataset” Tab representing the positive and negative samples

A web server offers a quick and simple way to do computational analysis. Additionally, the availability of such internet resources aids scholars in any upcoming breakthroughs. The m1A-Ensem, a free online web server for the suggested model, was created with this objective in mind and is accessible at https://taseersuleman-m1a-ensem1.streamlit.app/. It has four tabs including “*Home*”, “*Predictor*”, “*Dataset*” and “*Citations*”. The “*Home*” tab contains the m1A prediction model description. Figure 8 represents the screenshot of the webserver for the proposed model. The “*Predictor*” tab contains the sample sequence and input area. A user can input any length of sequence in the Input area. Figure 9 shows the “*Predictor*” tab with “Example” sequence button and Input area. The user has to click “submit” button and the result generated for each *Adenosine* (A) site as it is m1A site or non-m1A site. Figure 10 represent a sequence showing their actual position within the sequence and their status (m1A site of non-m1A site). Similarly, the “*Dataset*” tab contains the dataset samples used for training and testing the models. Figure 11 depicts the “*Dataset*” image.

## Conclusion

This study focused on detecting one of the most common post-transcriptional modifications, 1-methyladenosine (m1A), in RNA sequences using ensemble methods. Identifying m1A sites is crucial as this modification is associated with various human diseases, including mitochondrial respiratory chain defects, neurodevelopmental regression, X-linked intractable epilepsy, and obesity. A novel feature extraction mechanism was developed, taking into account both the positional and compositional attributes of nucleotides within RNA sequences. By calculating statistical moments, feature dimensionality reduction was achieved, streamlining the analysis. The resulting feature set was used to train several ensemble models based on stacking, bagging, and boosting techniques. The trained models underwent evaluation through cross-validation and independent testing. Performance was assessed using well-known accuracy metrics such as accuracy, sensitivity, specificity, and Matthew's correlation coefficient. Based on the best-performing ensemble model, the proposed model, m1a-ensem, was constructed. A comparative analysis of m1A-Ensem was conducted against existing predictors to gauge its effectiveness. The results demonstrated that m1A-Ensem outperformed other predictors in all accuracy metrics. Consequently, it can be concluded that the proposed model successfully enhanced the ability to identify modified m1A sites by employing the techniques described above. In summary, the research developed a novel approach to detect m1A sites in RNA sequences, which has implications for understanding and potentially treating various human diseases. By incorporating ensemble methods and a unique feature extraction mechanism, the m1A-Ensem model demonstrated superior performance in comparison to existing predictors, highlighting its potential for further applications in this field.

## Data Availability

The data and code of the current research study is available at https://github.com/taseersuleman/m1A-ensem-model.
